# Geriatric Knowledge Gaps of Community-Based Providers: A National Study of Asked Questions

**DOI:** 10.1093/geroni/igab046.3014

**Published:** 2021-12-17

**Authors:** Lee Lindquist, Aylin Madore, Stephanie Miller, Alice Kerr, Sara Bradley

**Affiliations:** 1 Northwestern University Feinberg School of Medicine, Chicago, Illinois, United States; 2 DBC Pri-Med, LLC, Boston, Massachusetts, United States; 3 Northwestern University Feinberg School of Medicine, Northwestern University Feinberg School of Medicine, Illinois, United States

## Abstract

Primary care providers (PCP) - internists, family practitioners, nurse practitioners, physician assistants - play an integral role in the care of older adults, although many receive limited geriatrics education. We sought to examine what questions community-based PCPs had about geriatrics and clinical care of older adults. As part of large clinical continuing medical education (CME) conferences across 12 states (FL,GA,CA,IL,NY,MA,DC, PA,AZ,TX,TN,WA), PCPs attended a live in-person 60-minute geriatrics-focused lecture and entered questions into a mobile application. Questions were then qualitatively analyzed using constant-comparison and tie-break methodology. At all sites, 103 questions were asked with 158 upticks (PCPs could check off that they had similar question) with a range of 3-18 questions per lecture. PCPs asked questions on the following common themes: 1.) Medication-related (e.g. discontinuing medicines in asymptomatic patients, optimizing pain relief), 2.) Dementia (e.g. prevention, nutraceuticals, agitation) 3.) Medicare Coding 4.) Falls 5.) Weight loss, and 6.) Insomnia. There were a number of questions referencing incorrect practices (e.g. prescribing inappropriate medications such as benzodiazepines for sleep, placement of gastric tubes in late-stage dementia, antibiotics to treat asymptomatic bacteria). In conclusion, community-based PCPs nationally experience gaps in geriatric knowledge and several utilize practices that could jeopardize older adult health. While attending CME-based lectures is one means of overcoming these gaps, some PCPs may not find time or realize geriatrics as an educational need. PCPs need to be better supported with opportunities to ask geriatric care-related questions in order to improve the care of older adults.

